# Skating techniques of ladies' world-class long-distance speed skaters to shorten curved-section time during the official 3,000 m race

**DOI:** 10.3389/fspor.2024.1396219

**Published:** 2024-05-23

**Authors:** Yuya Kimura, Toshiharu Yokozawa

**Affiliations:** Department of Sport Science and Research, Japan Institute of Sports Sciences, Tokyo, Japan

**Keywords:** centripetal acceleration, curvature radius, velocity, inclination of the body, elite athletes, international competition

## Abstract

This study aimed to identify the factors contributing to expedited passage through curved sections in skating by analyzing centripetal acceleration and skating motions during curving in a 3,000 m race for ladies' world-class speed skating. It included 14 elite skaters participating in the ladies' 3,000 m race held during the World Cup. The recorded area consisted of the first inner curve lane. Skaters were recorded as they passed through the measurement range at the initial, middle, and final stages of the race. Three synchronized high-speed cameras were used to record skaters from the front, back, and side. From the images obtained by the high-speed camera, 21 body endpoints and 4 blade edges were digitized at 50 Hz using specialized digitizing software. Three-dimensional coordinates of the 25 points were obtained using a panning direct linear transformation technique. The stroke-averaged centripetal acceleration and kinematic parameters were calculated based on the three-dimensional coordinates of the body during the curve-skating motion. Centripetal acceleration had a significant effect on the curved-section time in all three race stages (initial: *F* = 17.19, middle: *F* = 23.30, final: *F* = 16.64) and significantly decreased as the race progressed (left: *F *= 9.42, right: *F* = 8.05). Throughout the race, the right and left shanks and the body's center of mass (CM) during the stroke were raised (shank angle: left: *F* = 13.62, right: *F* = 11.02, CM height: left: *F* = 21.15, right: *F* = 21.69). The body-tilt angle for both strokes and shank-tilt angle for the right stroke were significantly correlated with centripetal acceleration in all race stages (body-tilt: left: initial: *r* = 0.80, middle: *r* = 0.75, final: *r* = 0.89, right: initial: *r* = 0.78, middle: *r* = 0.84, final: *r* = 0.67, right shank-tilt initial: *r* = 0.80, middle: *r* = 0.77, final: *r* = 0.63). These results suggested that to reduce the skating time through curved sections, maintaining an inward body tilt and minimizing the decrease in centripetal acceleration even in the final race stage are crucial considerations. They also suggested that when leaning the body inward and maintaining centripetal acceleration, the right shank should be leaned inward for the right stroke and the left shank should be leaned inward for the left stroke, or the left blade should be positioned farther to the right of the CM.

## 1 Introduction

Speed skating is a competition in which skaters compete to skate a given distance in the shortest possible time around a 400 m track, which consists of two straight and two curved sections. In the double-track race, two skaters skate on 4 m wide inner and outer lanes and switch from one lane to the other in each lap. For C-type tracks, i.e., the most commonly used tracks in official competitions, the inner course lines for curved sections are set at radii of 26 m and 30 m for the inner and outer lanes, respectively. In medium- and long-distance speed-skating events, both the skating velocity and excess distance [i.e., difference between the shortest distance and the distance actually skated (mostly due to curved sections in the track)] have been reported as important factors that can affect official times and rankings (1). The excess distance covered in the curved sections may be attributed to the centrifugal force acting on the skater's body, which makes moving around an arc of the same radius as that of the inner line of the curve difficult. Thus, to skate through a curved section in a shorter time, skaters must attain a higher skating velocity and minimize the radius of the arc. To achieve these two objectives, the skater must increase centripetal acceleration, which is calculated by squaring the skating velocity and dividing it by the curvature radius. Therefore, to identify better curve-skating techniques, centripetal acceleration (composed of the skating velocity and curvature radius) should be used as a performance index and as a value to relate to other kinematic parameters.

After the introduction of klap skating, kinematic investigations of curve skating by world-class skaters have been conducted for men's 5,000 m and 1,500 m races to identify the techniques for achieving a higher skating velocity (2, 3). In addition, Yuda et al. (4) measured the reaction force applied to the left blade during curve skating and found that the horizontal component of the reaction force had a profound effect on the skating velocity. However, most of these studies have examined the skating velocity as a performance index; no studies have involved detailed analyses of the centripetal acceleration in this regard. In addition, several previous studies measured the reaction forces applied to the left and right blades during curve skating (5–9), but none of them examined the relationship with the centripetal acceleration or performance. Furthermore, reports on ladies’ long-distance races are fewer than those on men's races. Although a study on straight skating in ladies' long-distance races has been reported (10–13), no report has been published on curve skating in ladies’ long-distance races.

Previous relevant studies have revealed the following: (1) the horizontal component of the reaction force applied to the left blade decreases in the latter half of the race and affects the skating velocity (4); (2) world-class skaters in men's 5,000 m races tilt their bodies and shanks more inward than junior skaters do (14); and (3) skaters with higher skating velocities in men's 1,500 m races tilt their bodies more inward during the right stroke (2). Based on these findings, we hypothesized that to shorten skating time through curved sections in ladies' 3,000 m races, skaters must attain higher levels of centripetal acceleration than their current performance. We also hypothesized that the centripetal acceleration would decrease and the inward tilt of the body and supporting leg shank would become shallower as the race progresses. Thus, in the present study, we aimed to identify the factors necessary for skating through curved sections in a shorter time by analyzing the centripetal acceleration and skating motions during curving in a 3,000 m race for ladies' world-class speed skating.

## 2 Materials and methods

### 2.1 Data collection

This observational study included skaters participating in the ladies' 3,000 m races held during the 2019–2020 International Skating Union Speed Skating World Cup. Permission to record a video was requested in advance and obtained from the organizing committee through the Japan Skating Federation. This study was approved by the Ethical Review Committee of the Japan Institute of Sports Sciences (2019-030).

**Figure 1:**
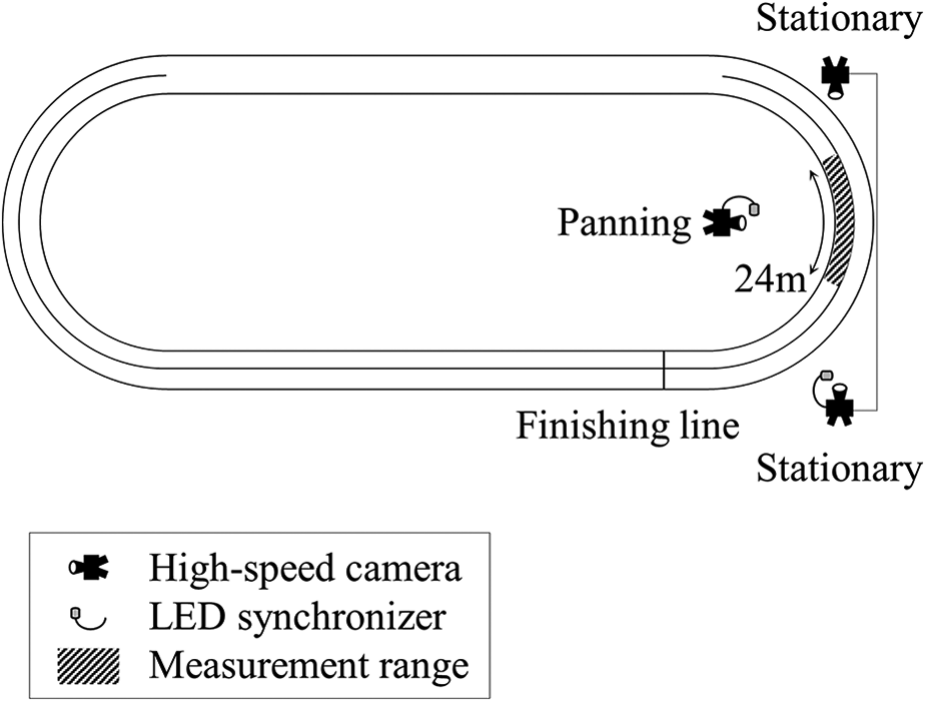
Setup for three-dimensional recording. The positions of the high-speed camera and LED synchronizer are shown. The diagonal lined part indicates the measurement range.

The measurement range consisted of a 4 m wide, 24 m long, and 1.25 m high area of the first inner curve lane. Three high-speed cameras (Phantom VEO 410S and MIRO LC111, Vision Research, USA) were used to record skaters from the front, back, and side. The front and back cameras were stationary, while the side camera was panned to track the skaters. Skating motion was captured at 300 Hz with an exposure time of 1/1,000 s (Figure 1). Videos were synchronized using a wireless LED synchronizer (PTS-168, DKH, Japan). In the 3,000 m race, skaters started near the entrance to the second curve, diagonally opposite the finishing line, and completed seven and a half laps. In this study, the first lap was defined as a half lap from the start of the race to the first crossing of the finishing line; the subsequent laps were defined as the second through the eighth laps. Data were captured at the third, fifth, and seventh laps (corresponding to distances of approximately 650 m, 1,450 m, and 2,250 m, respectively) for skaters starting in the outer lane and at the fourth, sixth, and eighth laps (corresponding to distances of approximately 1,050 m, 1,850 m, and 2,650 m, respectively) for skaters starting in the inner lane. These laps comprised the initial, middle, and final stages of the race, respectively. In addition, a digital video camera (W870M, Panasonic, Japan) was used to record the skaters during their races from the top row of the spectator stand. The skaters were recorded at 60 Hz with an exposure time of 1/500 s. Among the skaters with good official times, 14 skaters who were recorded without deficiencies were included in the analyses. Their mean age, height, body mass, and official time were 27.57 ± 6.21 years, 1.68 ± 0.07 m, 60.36 ± 6.80 kg, and 246.39 ± 3.08 s, respectively.

### 2.2 Data analysis

From the images obtained by the digital video camera, frames in which either the left or the right blade tip passed through the entrance and exit of the first inner curve lane were extracted, and the curved-section times required in the initial, middle, and final stages of the race were calculated (15).

From the images obtained by the high-speed camera, 21 body endpoints and 4 blade edges were digitized at 50 Hz using specialized digitizing software (Frame-Dias VI, DKH, Japan) (13); the anatomical points were manually identified on the high-speed camera images. Three-dimensional coordinates of the 25 points were obtained using a panning direct linear transformation technique; these were smoothed using a fourth-order Butterworth low-pass digital filter with a cut-off of 2.5–7.0 Hz (determined using a residual method) (16). Standard errors in the constructed coordinates of the control points were 0.014 m (*x*-axis), 0.012 m (*y*-axis), and 0.011 m (*z*-axis). In this study, “blade-off” was defined as the moment at which the entire bottom portion of the blade left the surface of the ice, and “blade-on” was defined as the moment at which the bottom of the blade made even partial contact with the surface of the ice. Both were detected with a resolution of 300 Hz. In addition, “left stroke” was defined as the period from right blade-off to left blade-off, and “right stroke” was defined as the period from left blade-off to right blade-off (13). Two consecutive strokes at the initial, middle, and final stages of the race were analyzed in this study. The time taken for one stroke was defined as the stroke time. The strokes were divided into single- and double-support phases on the basis of the opposite-side blade-on (2), and the time required for each phase was calculated. The coordinates of the body's center of mass (CM) were calculated using the body segment inertial parameters for female speed skaters provided by Yokozawa et al. (17). The direction tangential to the course at the position of the CM was considered the direction of travel, and the distance the CM moved in that direction during a stroke was defined as the stroke length. The directional velocity was calculated by dividing the stroke length by the stroke time. The curvature radius (*R*) was calculated as follows:

$$R\;=\;V_{\mathrm{start}}^{3}/\;|\;V_{\mathrm{start}}\times \;[\;(V_{\mathrm{end}}-\;V_{\mathrm{start}})\;/\;T\;]\;|$$

Here, *T* is the stroke time, and *V*_start_ and *V*_end_ are the CM horizontal velocity vectors at the beginning and end of the stroke, respectively. Furthermore, the centripetal acceleration was calculated by dividing the square of the CM horizontal velocity by the curvature radius.

**Figure 2:**
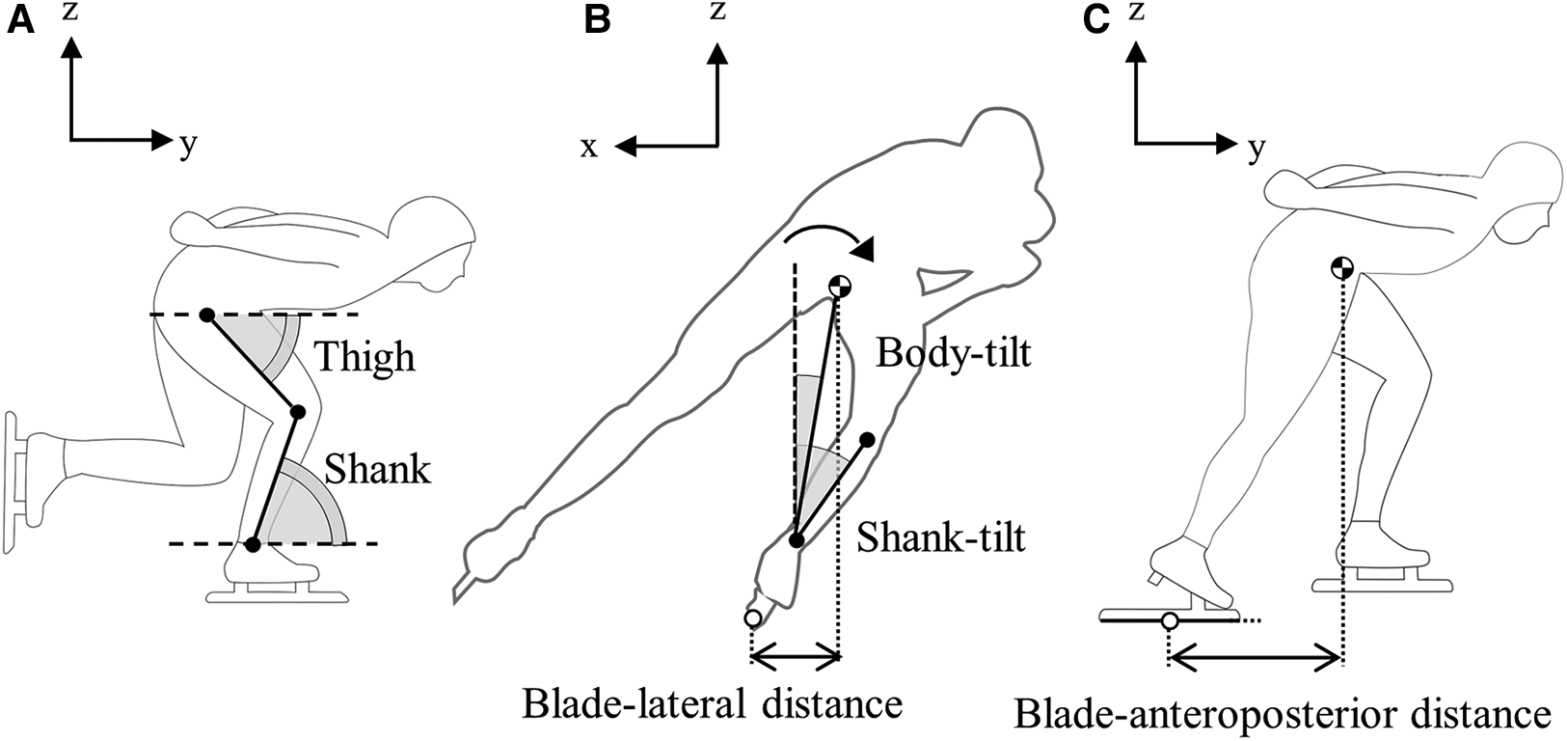
Definitions of kinematic parameters. (**A**) Thigh and shank angles, (**B**) body- and shank-tilt angles and blade-lateral distance, and (**C**) blade-anteroposterior distance. The cross-shaped circle indicates the position of the whole-body center of mass.

A local coordinate system was set up such that the horizontal component of the CM velocity was the *y*-axis, the vertical upward direction was the *z*-axis, and the cross product of the *y*-axis and *z*-axis was the *x*-axis. The CM height was defined as the *z* component of the CM coordinates and was normalized by height. The thigh and shank angles were defined as the angles between the thigh and shank segments and the *y*-axis on the *y*-*z* plane, respectively (Figure 2A) (13). On the *x*-*z* plane, the body tilt angle was defined as the angle between the vector from the ankle joint to the CM and the *z*-axis. Similarly, the shank tilt angle was defined as the angle between the shank segment and the *z*-axis. Positive values for the body and shank-tilt angles indicated a tilt toward the center of the curve (Figure 2B) (14). The CM height, body-tilt angle, and shank-tilt angle were averaged for each stroke. The thigh and shank angles were calculated at the beginning of the stroke, the beginning of the double-support phase, and the end of the stroke.

The blade-anteroposterior distance was calculated from the difference between the *y*-coordinate of the CM and the *y*-coordinate of the midpoint at both ends of the blade at blade-off (Figure 2C) (2). The blade lateral distance was calculated from the difference between the *x*-coordinate of the CM and the *x*-coordinate of the midpoint at both ends of the blade at blade-on (Figure 2B).

Differences in each parameter among the race stages (initial, middle, and final) were analyzed to confirm the changes occurring as the race progressed. Additionally, the relationships between the official time and curved-section time, curved-section time and centripetal acceleration of the left and right strokes, and centripetal acceleration of the left and right strokes and directional velocity and curvature radius at the same stroke were examined to explore the factors related to performance. Furthermore, the relationship between each parameter and centripetal acceleration in the same race stage was confirmed, and the skating motion characteristics of skaters with high centripetal acceleration were examined.

### 2.3 Statistical analysis

The mean value and standard deviation of each parameter were calculated and are listed in the tables. To confirm the normality of the respective data, the Shapiro-Wilk test was performed, and the following statistical methods were performed on the parameters exhibiting normality. Differences in each parameter among the race stages (initial, middle, and final) were examined using one-way repeated-measures analysis of variance (ANOVA). If the main effect was significant, *post-hoc* analysis was performed using Bonferroni multiple comparisons. Furthermore, multiple regression analysis was performed with the curved-section time as the dependent variable and the centripetal acceleration of the left and right strokes in the same race stage as the independent variables; the analysis was also performed with the centripetal acceleration as the dependent variable and the directional velocity and curvature radius at the same stroke as the independent variables. Pearson's product-moment correlation coefficients were calculated to examine the relationships between the official time and curved-section time and between each parameter and the centripetal acceleration in the same race stage. The significance level was set at 5%. The sample size for this study was 14. For Pearson's product-moment correlation coefficient, an effect size of *r* ≥ 0.63, which is a power of 80%, was considered large and is listed in the table. Similarly, for multiple regression analysis, an effect size of *R*^2^ ≥ 0.40, corresponding to 80% power, was considered large and is also presented in the table. All statistical analyses were performed using SPSS Statistics (ver. 24, IBM, USA).

## 3 Results

### 3.1 Performance descriptors

The curved-section time was the longest in the final stage, followed by the middle and initial stages (Table 1). In the middle and final stages, the curved-section time exhibited a significant positive relationship with the official time. The centripetal acceleration for both the left and right strokes was significantly smaller in the middle and final stages than in the initial stage (Table 2). Multiple regression analysis yielded a significant standardized partial regression coefficient between the curved-section time and centripetal acceleration of the left and right strokes for all race stages. The model of the curved-section time (*Y*) and the centripetal acceleration of the left and right strokes (left: *X*_1_, right: *X*_2_) during each race stage are described below.

$$Y\;=\;8.73\;-\;0.20X_{1}-\;0.15X_{2}\;(\mathrm{initial})$$

$$Y\;=\;9.18\;-\;0.21X_{1}-\;0.20X_{2}\;(\mathrm{middle})$$

$$Y\;=\;10.21\;-\;0.21X_{1}-\;0.37X_{2}\;(\mathrm{final})$$

**Table 1:** Mean values and results of analysis of variance for the curved-section time in the initial, middle, and final stages of the race (labeled initial, middle, and final, respectively) and correlations of the curved-section time with the official time (r. OT).

		Mean	S.D.	r. OT	95% CI	*p*	Power	Main effect		Significant difference
					* *	* *		*F*	*p*	(Race phases)
Curved-section time (s)	Initial	6.62	0.10	0.31	−0.26 to 0.72	.279	0.20	22.90	<.001	Initial<<Middle<<Final Initial<<<Final
	Middle	6.78	0.14	0.75^a^	0.37–0.92	.002	0.97			
	Final	6.92	0.17	0.71^a^	0.29–0.90	.004	0.93			

>>>: *p* < 0.001, >>: *p* < 0.01, >: *p* < 0.05.

^a^ Effect size: large.

**Table 2:** Mean values and results of analysis of variance for centripetal acceleration, directional velocity, and curvature radius in the initial, middle, and final stages of the race and results of multiple regression analysis.

Dependent variable: curved-section time (s) Independent variable: left stroke Centripetal acceleration, right stroke centripetal acceleration (m/s^2^)																	
		Left: Centripetal acceleration (m/s^2^)					Right: centripetal acceleration (m/s^2^)										
		Mean	S.D.	S.P.R.C	95% CI	*p*	Mean	S.D.	S.P.R.C	95% CI	*p*	DW	VIF	R^2^	F	*p*	Power
Initial		5.81	0.50	−1.04	−0.29 to −0.15	<.001	6.25	0.32	−0.50	−0.29 to −0.07	.018	1.66	1.46	0.71^a^	17.19	<.001	0.99
Middle		5.40	0.37	−0.57	−0.29 to −0.07	.001	5.94	0.50	−0.79	−0.31 to −0.14	<.001	2.70	1.03	0.77^a^	23.30	<.001	0.99
Final		5.31	0.48	−0.57	−0.34 to −0.10	.003	5.85	0.34	−0.73	−0.53 to −0.20	<.001	1.86	1.02	0.71^a^	16.64	<.001	0.99
Main effect	*F*	9.42					8.05										
	*p*	<.001					.002										
Significant difference (race phases)		Initial>>Middle, Initial>Final					Initial>Middle, Initial>>Final										
Dependent variable: left stroke centripetal acceleration (m/s^2^) Independent variable: left stroke directional velocity (m/s), left stroke Curvature radius (m)																	
		Left: directional velocity (m/s)					Left: curvature radius (m)										
		Mean	S.D.	S.P.R.C	95% CI	*p*	Mean	S.D.	S.P.R.C	95% CI	*p*	DW	VIF	*R* ^2^	*F*	*p*	Power
Initial		12.41	0.22	0.28	0.16–1.19	.015	28.22	2.04	−0.88	−0.27 to −0.16	<.001	1.67	1.01	0.88^a^	47.79	<.001	1.00
Middle		12.02	0.39	0.48	0.24–0.71	<.001	28.97	1.78	−0.98	−0.26 to −0.16	<.001	2.23	1.13	0.86^a^	41.21	<.001	1.00
Final		11.85	0.40	0.48	0.36–0.82	<.001	28.30	2.24	−0.99	−0.25 to −0.17	<.001	2.01	1.09	0.91^a^	70.09	<.001	1.00
Main effect	*F*	13.76					1.81										
	*p*	<.001					.184										
Significant difference (race phases)		Initial>>Middle, Final					n.s.										
Dependent variable: right stroke centripetal acceleration (m/s^2^) Independent variable: right stroke directional velocity (m/s), right stroke Curvature radius (m)																	
		Right: directional velocity (m/s)					Right: curvature radius (m)										
		Mean	S.D.	S.P.R.C	95% CI	*p*	Mean	S.D.	S.P.R.C	95% CI	*p*	DW	VIF	R^2^	*F*	*p*	Power
Initial		12.37	0.33	0.49	0.18–0.76	.004	26.14	1.59	−1.04	−0.27 to −0.15	<.001	3.15	1.29	0.81^a^	29.54	<.001	0.99
Middle		12.10	0.26	0.38	0.04–1.45	.040	26.28	1.76	−0.61	−0.28 to −0.07	.003	1.54	2.14	0.84^a^	34.40	<.001	1.00
Final		11.85	0.30	0.70	0.50–1.14	<.001	25.55	1.26	−0.78	−0.29 to −0.14	<.001	1.93	1.06	0.81^a^	29.22	<.001	0.99
Main effect	*F*	10.23					1.44										
	*p*	<.001					.256										
Significant difference (race phases)		Initial>>Final, Middle>Final					n.s.										

>>>: *p* < 0.001, >>: *p* < 0.01, >: *p* < 0.05.

n.s., no significant; S.P.R.C., standardized partial regression coefficient; DW, Durbin-Watson ratio; VIF, variance inflation factor; *R*^2^, determination coefﬁcient adjusted for the degrees of freedom.

^a^ Effect size: large.

The determination coefﬁcient adjusted for the degrees of freedom (*R*^2^) for the respective models was 0.71, 0.77, and 0.71 for the initial, middle, and final stages, respectively.

The directional velocity of the left stroke was significantly smaller in the middle and final stages than in the initial stage (Table 2). The directional velocity of the right stroke was significantly smaller in the final stage than in the initial and middle stages. Multiple regression analysis yielded a significant standardized partial regression coefficient between the centripetal acceleration and directional velocity and between the centripetal acceleration and curvature radius for both the left and right strokes and for all race stages. The model of the centripetal acceleration (*Y*) and directional velocity (*X*_1_) and curvature radius (*X*_2_) of the left stroke during each race stage are described below.

$$Y\;=\;3.57\;+\;0.67X_{1}-\;0.22X_{2}\;(\mathrm{initial})$$

$$Y\;=\;5.68\;+\;0.47X_{1}-\;0.21X_{2}\;(\mathrm{middle})$$

$$Y\;=\;4.27\;+\;0.59X_{1}-\;0.21X_{2}\;(\mathrm{final})$$

The determination coefﬁcient adjusted for the degrees of freedom (*R*^2^) for the respective models was 0.88, 0.86, and 0.91 for the initial, middle, and final stages, respectively. The model of the centripetal acceleration (*Y*) and directional velocity (*X*_1_) and curvature radius (*X*_2_) of the right stroke during each race stage are described below.

$$Y\;=\;5.84\;+\;0.47X_{1}-\;0.21X_{2}\;(\mathrm{initial})$$

$$Y\;=\;1.52\;+\;0.74X_{1}-\;0.17X_{2}\;(\mathrm{middle})$$

$$Y\;=\;1.59\;+\;0.82X_{1}-\;0.21X_{2}\;(\mathrm{final})$$

The determination coefﬁcient adjusted for the degrees of freedom (*R*^2^) for the respective models was 0.81, 0.84, and 0.81 for the initial, middle, and final stages, respectively.

The stroke length for the left stroke was significantly smaller in the middle and final stages than in the initial stage (Table 3). The double-support phase time for the right stroke was significantly longer in the middle and final stages than in the initial stage.

**Table 3:** Mean values and results of analysis of variance for the stroke parameters in the initial, middle, and final stages of the race and correlations of these parameters with centripetal acceleration (r. CA).

			Mean	S.D.	r. CA	95% CI	*p*	Power	Main effect		Significant difference
							* *		*F*	*p*	(Race phases)
Left	Stroke time (s)	Initial	0.53	0.04	−0.39	−0.76 to 0.18	.174	0.31	0.69	.511	n.s.
		Middle	0.52	0.04	0.39	−0.18 to 0.76	.168	0.31			
		Final	0.52	0.04	−0.11	−0.61 to 0.45	.712	0.07			
	Single-support phase (s)	Initial	0.40	0.03	−0.42	−0.78 to 0.14	.134	0.36	0.59	.560	n.s.
		Middle	0.39	0.03	0.42	−0.14 to 0.78	.132	0.36			
		Final	0.40	0.04	−0.13	−0.62 to 0.43	.652	0.07			
	Double-support phase (s)	Initial	0.13	0.02	0.00	−0.53 to 0.53	.988	0.05	0.71	.500	n.s.
		Middle	0.13	0.02	0.14	−0.42 to 0.62	.630	0.08			
		Final	0.12	0.02	−0.01	−0.54 to 0.52	.986	0.05			
	Stroke length (m)	Initial	6.56	0.40	−0.33	−0.73 to 0.24	.254	0.23	5.55	.009	Initial>Middle, Final
		Middle	6.23	0.53	0.39	−0.18 to 0.76	.164	0.31			
		Final	6.20	0.56	−0.03	−0.55 to 0.51	.924	0.05			
Right	Stroke time (s)	Initial	0.58	0.04	−0.22	−0.67 to 0.35	.445	0.12	1.74	.200	n.s.
		Middle	0.59	0.05	−0.45	−0.79 to 0.11	.109	0.41			
		Final	0.59	0.05	−0.32	−0.73 to 0.25	.271	0.21			
	Single-support phase (s)	Initial	0.46	0.03	−0.40	−0.77 to 0.17	.160	0.32	0.22	.810	n.s.
		Middle	0.46	0.04	−0.51	−0.82 to 0.03	.064	0.53			
		Final	0.46	0.04	−0.51	−0.82 to 0.03	.061	0.53			
	Double-support phase (s)	Initial	0.12	0.03	0.18	−0.39 to 0.65	.527	0.10	7.24	.003	Initial<Middle Initial<<Final
		Middle	0.13	0.02	−0.09	−0.59 to 0.46	.756	0.06			
		Final	0.14	0.03	0.18	−0.39 to 0.65	.538	0.10			
	Stroke length (m)	Initial	7.11	0.40	−0.29	−0.71 to 0.28	.315	0.18	0.79	.470	n.s.
		Middle	7.14	0.56	−0.26	−0.70 to 0.31	.374	0.15			
		Final	7.01	0.58	−0.17	−0.64 to 0.40	.562	0.09			

>>>: *p* < 0.001, >>: *p* < 0.01, >: *p* < 0.05, n.s., no significant.

### 3.2 Kinematics

The CM height for both the left and right strokes was significantly higher in the middle and final stages than in the initial stage (Table 4). The body-tilt angle for the left stroke and shank-tilt angle for the right stroke were significantly smaller in the middle and final stages than in the initial stage. The body-tilt angle for the right stroke and shank-tilt angle for the left stroke were significantly smaller in the middle stage than in the initial stage. The blade-anteroposterior distance for the right stroke was significantly greater in the final stage than in the initial stage. At all race stages, the body-tilt angles for both strokes and the shank-tilt angle for the right stroke exhibited a significant positive correlation with the centripetal acceleration. For the right stroke, the CM height exhibited a significant negative correlation with the centripetal acceleration at all race stages.

**Table 4:** Mean values and results of analysis of variance for the blade-anteroposterior and -lateral distances, CM height, and body- and shank-tilt angles in the initial, middle, and final stages of the race and correlations of these parameters with centripetal acceleration (r. CA).

			Mean	S.D.	r. CA	95% CI	*p*	Power	Main effect		Significant difference
						* *	* *		*F*	*p*	(Race phases)
Left	Blade-anteroposterior distance (m)	Initial	0.37	0.05	−0.02	−0.55 to 0.52	.955	0.05	1.37	.270	n.s.
		Middle	0.38	0.04	−0.20	−0.66 to 0.37	.488	0.11			
		Final	0.39	0.05	−0.28	−0.71 to 0.29	.325	0.17			
	Blade-lateral distance (m)	Initial	−0.21	0.04	−0.20	−0.66 to 0.37	.489	0.11	0.31	.740	n.s.
		Middle	−0.20	0.04	−0.38	−0.76 to 0.19	.179	0.29			
		Final	−0.21	0.04	−0.30	−0.72 to 0.27	.299	0.19			
	CM height (no unit)	Initial	0.38	0.01	0.27	−0.30 to 0.70	.347	0.16	21.15	<.001	Initial<<Middle Initial<<<Final
		Middle	0.39	0.02	0.10	−0.46 to 0.60	.730	0.06			
		Final	0.40	0.01	−0.12	−0.61 to 0.44	.684	0.07			
	Body-tilt angle (deg)	Initial	28.00	1.95	0.80^a^	0.47 to 0.93	.001	1.00	6.40	.007	Initial>Middle, Final
		Middle	26.79	1.25	0.75^a^	0.36 to 0.92	.002	0.97			
		Final	26.37	1.86	0.89^a^	0.68 to 0.96	.000	1.00			
	Shank-tilt angle (deg)	Initial	34.58	2.94	0.52	−0.02 to 0.82	.054	0.55	6.17	.006	Initial>Middle
		Middle	32.88	2.75	0.14	−0.42 to 0.62	.634	0.08			
		Final	32.54	3.18	0.36	−0.21 to 0.75	.209	0.26			
Right	Blade-anteroposterior distance (m)	Initial	0.24	0.05	−0.15	−0.63 to 0.41	.603	0.08	4.43	.020	Initial<Final
		Middle	0.26	0.05	−0.19	−0.66 to 0.38	.517	0.10			
		Final	0.27	0.06	0.22	−0.35 to 0.67	.458	0.12			
	Blade-lateral distance (m)	Initial	0.23	0.05	0.26	−0.31 to 0.69	.377	0.15	0.04	.960	n.s.
		Middle	0.23	0.04	0.38	−0.19 to 0.76	.175	0.29			
		Final	0.23	0.04	0.24	−0.33 to 0.68	.418	0.14			
	CM height (no unit)	Initial	0.39	0.01	−0.82^a^	−0.94 to −0.51	.000	1.00	21.69	<.001	Initial<<Middle Initial<<<Final
		Middle	0.39	0.02	−0.81^a^	−0.94 to −0.49	.000	1.00			
		Final	0.40	0.01	−0.62	−0.87 to −0.13	.018	0.77			
	Body-tilt angle (deg)	Initial	32.18	1.79	0.78^a^	0.43–0.93	.001	0.99	3.61	.040	Initial>Middle
		Middle	30.97	2.53	0.84^a^	0.56–0.95	.000	1.00			
		Final	31.19	1.45	0.67^a^	0.22–0.89	.009	0.87			
	Shank-tilt angle (deg)	Initial	28.85	3.55	0.80^a^	0.47–0.93	.001	1.00	6.77	.004	Initial>Middle, Final
		Middle	27.26	3.66	0.77^a^	0.40–0.92	.001	0.99			
		Final	27.09	2.56	0.63^a^	0.15–0.87	.015	0.80			

>>>: *p* < 0.001, >>: *p* < 0.01, >: *p* < 0.05, n.s., no significant.

^a^ Effect size: large.

The thigh angle at the beginning of the left stroke was significantly greater in the middle and final stages than in the initial stage (Table 5). The thigh and shank angles at the beginning of the double-support phase for the left stroke were significantly greater in the final stage than those in the initial stage. The shank angle at the beginning of the double-support phase for the right stroke was significantly greater in the final stage than in the initial and middle stages. In the initial stage, the thigh angle at the end of the left stroke exhibited a significant positive relationship with centripetal acceleration and a significant negative relationship in the final stage. In the initial stage, the thigh angles at the beginning of the double-support phase for the right stroke and at the end of the right stroke exhibited a significant negative relationship with the centripetal acceleration. In the initial and middle stages, the shank angle at the beginning of the double-support phase for the right stroke exhibited a significant negative relationship with the centripetal acceleration.

**Table 5:** Mean values and results of analysis of variance for the thigh/shank angles at the beginning of the stroke, beginning of the double-support phase, and end of the stroke in the initial, middle, and final stages of the race and correlations of these parameters with centripetal acceleration (r. CA).

		(deg)		Mean	S.D	r. CA	95% CI	*p*	Power	Main effect		Significant difference
							* *	* *		*F*	*p*	(Race phases)
Left	Thigh	Beginning of the stroke	Initial	29.04	3.31	0.22	−0.35 to 0.67	.443	0.12	6.92	.004	Initial<Middle, Final
			Middle	31.44	4.53	0.12	−0.44 to 0.61	.674	0.07			
			Final	32.04	3.86	0.41	−0.15 to 0.77	.141	0.34			
		Beginning of the double-support phase	Initial	58.41	7.38	0.19	−0.38 to 0.65	.514	0.10	6.65	.010	Initial<Final
			Middle	62.48	7.31	−0.03	−0.55 to 0.51	.916	0.05			
			Final	64.20	7.67	−0.14	−0.62 to 0.42	.626	0.08			
		End of the stroke	Initial	91.87	6.18	0.76^a^	0.76 to 0.91	.002	0.98	0.33	.720	n.s.
			Middle	92.72	5.72	0.08	−0.47 to 0.59	.783	0.06			
			Final	91.97	5.21	−0.61	−0.86 to −0.12	.021	0.75			
	Shank	Beginning of the stroke	Initial	63.82	4.77	0.17	−0.40 to 0.64	.559	0.09	1.37	.270	n.s.
			Middle	64.50	5.24	0.03	−0.51 to 0.55	.919	0.05			
			Final	64.98	4.19	−0.16	−0.64 to 0.41	.588	0.09			
		Beginning of the double-support phase	Initial	50.26	6.13	0.13	−0.43 to 0.62	.646	0.07	13.62	<.001	Initial<<<Final
			Middle	52.38	5.82	0.11	−0.45 to 0.61	.709	0.07			
			Final	54.39	4.94	−0.09	−0.59 to 0.46	.750	0.06			
		End of the stroke	Initial	58.92	7.11	0.37	−0.20 to 0.75	.198	0.28	1.32	.280	n.s.
			Middle	57.93	6.99	0.30	−0.27 to 0.72	.295	0.19			
			Final	59.74	7.05	0.30	−0.27 to 0.72	.291	0.19			
Right	Thigh	Beginning of the stroke	Initial	36.02	4.63	−0.04	−0.56 to 0.50	.899	0.05	3.58	.040	n.s.
			Middle	37.47	4.25	−0.21	−0.67 to 0.36	.467	0.11			
			Final	38.52	4.13	−0.21	−0.67 to 0.36	.481	0.11			
		Beginning of the double-support phase	Initial	67.37	7.42	−0.61	−0.86 to −0.12	.022	0.75	1.13	.340	n.s.
			Middle	66.16	5.51	−0.35	−0.74 to 0.22	.219	0.25			
			Final	68.03	6.61	−0.47	−0.80 to 0.08	.091	0.45			
		End of the stroke	Initial	82.91	6.75	−0.57	−0.85 to −0.06	.034	0.66	1.74	.200	n.s.
			Middle	82.00	8.55	−0.28	−0.71 to 0.29	.331	0.17			
			Final	84.35	10.96	−0.07	−0.58 to 0.48	.801	0.06			
	Shank	Beginning of the stroke	Initial	67.61	3.74	0.07	−0.48 to 0.58	.820	0.06	3.43	.050	n.s.
			Middle	68.55	3.27	0.13	−0.43 to 0.62	.660	0.07			
			Final	68.87	3.21	0.30	−0.27 to 0.72	.294	0.19			
		Beginning of the double-support phase	Initial	59.86	5.17	−0.57	−0.85 to −0.06	.032	0.66	11.02	<.001	Initial<<Final Middle<Final
			Middle	60.79	5.32	−0.60	−0.86 to −0.10	.024	0.73			
			Final	63.19	5.08	−0.47	−0.80 to 0.08	.088	0.45			
		End of the stroke	Initial	69.52	6.32	−0.17	−0.64 to 0.40	.566	0.09	0.62	.550	n.s.
			Middle	70.48	5.11	0.00	−0.53 to 0.53	.991	0.05			
			Final	70.51	5.51	−0.23	−0.68 to 0.34	.437	0.13			

>>>: *p* < 0.001, >>: *p* < 0.01, >: *p* < 0.05, n.s., no significant.

^a^ Effect size: large.

### 3.3 Comparison of individual cases

**Figure 3:**
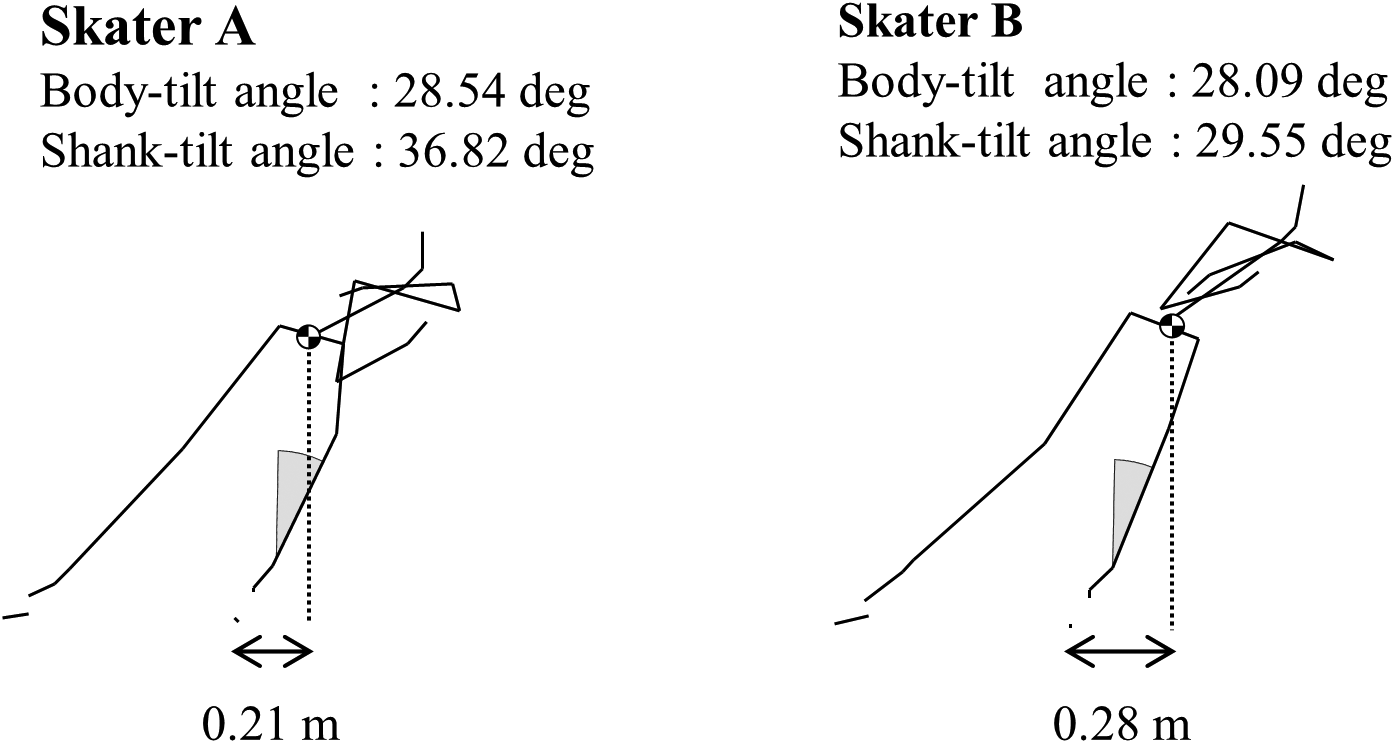
Stick pictures of skaters (**A**,**B**) at the beginning of the left stroke in the final stage of the race. The cross-shaped circle indicates the position of the whole-body center of mass. The double arrow indicates the lateral distance between the left-blade midpoint and the whole-body center of mass.

We compared the left strokes of skaters A and B in the final stage; these skaters reached the podium and had significantly different left shank-tilt angles (Figure 3). Both skaters tilted their bodies more inward, with skater A having the second largest angle and skater B having the third largest angle among the skaters in this study. In contrast, with respect to the left shank-tilt angle, skater A had the largest angle and skater B had a relatively smaller angle (12th largest) among the skaters in this study. Focusing on the blade-lateral distance, skater B had the largest value among the skaters in this study.

## 4 Discussion

In the present study, we aimed to identify the crucial variables shortening the skating time through a curved section by analyzing the centripetal acceleration and skating motions during curving in a 3,000 m race for ladies' world-class long-distance speed skating. We hypothesized that to shorten the skating time through curved sections, skaters in ladies' 3,000 m races must achieve greater centripetal acceleration. We also hypothesized that the centripetal acceleration would decrease and the inward tilt of the body and supporting leg shank would become shallower as the race progresses. Findings that supported our hypothesis included the following: (1) the centripetal acceleration greatly affected the required curved-section time (Table 2), (2) the centripetal acceleration decreased in the middle and final stages of the race (Table 2), and (3) skaters exerting a greater centripetal acceleration tilted their bodies and right shanks more inward (Table 4). Conversely, no significant correlation was identified between the shank-tilt angle and centripetal acceleration during the left stroke (Table 4). Most of our study findings aligned with our hypotheses, except for the relationship between the shank-tilt angle and centripetal acceleration during the left stroke.

### 4.1 Changes in the skating motion during the race

The centripetal acceleration and directional velocity decreased in the final stage for both strokes (Table 2). Considering that no significant changes were identified in the curvature radius as the race progressed, the decrease in the centripetal acceleration in the final stage may have affected the decrease in the directional velocity rather than taking a wider line in the curved lane.

In the final stage, the right-blade position became more backward relative to the CM at the end of the right stroke (Table 4). Yokozawa et al. (2) reported that in a men's 1,500 m race, skaters with higher velocities during curving and better official times had smaller blade-anteroposterior distances at the end of the right stroke, indicating that an effective push-off motion with the right blade as it transitioned toward the right side of the CM produced a large centripetal force in better skaters. Therefore, difficulties in the push-off motion to the right in the final stage may have influenced the decrease in the centripetal acceleration as the race progressed.

In the final stage, the CM height increased as the thigh and shank became more vertical (Tables 4, 5). In other words, a larger knee-joint angle resulted in a higher position of the CM as the race progressed. In the left stroke in curve skating, the exertion of large knee-joint extension torque is critical while keeping the knee joint flexed to increase the lateral component of the left blade-reaction force (4, 18). Therefore, the skaters in this study may have changed their push-off motions to make the exertion of large knee-joint extension torque more difficult as the race progressed.

### 4.2 Curve skating for a better performance

The excess distance, the difference between the shortest distance and the distance actually skated, is an important variable that can affect official times and rankings, mostly due to curved sections in the track (1). The results of the multiple regression analysis in this study revealed that better skaters were able to reduce the excess distance in the curved section with a larger centripetal acceleration (Table 2). Based on the finding that the centripetal acceleration affected the curved-section time, identification of the factors necessary to skate through the curved section in a shorter time seems possible by exploring the factors that contribute to centripetal acceleration. Therefore, we focused on the middle and final stages, during which a significant correlation was observed between the official time and curved-section time. We also considered suggestions for better performance based on the relationship between the centripetal acceleration and each parameter.

In the men's 5,000 m race, the world-class skaters tilted their bodies more inward than the junior skaters (14), and in the men's 1,500 m race, the skaters with higher skating velocities tilted their bodies more inward during the right stroke than those with lower skating velocities (2). The results of this study, which analyzed the ladies' 3,000 m race, showed that in the right stroke, skaters with a greater centripetal acceleration tilted their bodies and right shanks more inward (Table 4). To increase the centripetal acceleration, the body should be tilted more inward to balance the moment with the centrifugal force applied to the body. Better skaters were able to tilt their bodies inward by tilting their right shanks greatly inward. In the left stroke, those with a greater centripetal acceleration tilted their bodies more inward; however, no significant relationship was identified between the centripetal acceleration and left shank-tilt angle (Table 4). Tilting the body greatly inward was necessary for generating centripetal acceleration for the left stroke as well as the right stroke but not necessary for tilting the left shank greatly inward.

To delve further into the consequences of these results, we compared two of the top skaters (Figure 3) and found that both skaters tilted their bodies more inward during the left stroke. However, skater A also had a large left shank-tilt angle, suggesting that she was able to tilt her body inward by tilting her left shank greatly inward. On the other hand, skater B had a smaller left shank-tilt angle and a larger blade-lateral distance at the beginning of the stroke. This indicated that skater B placed the left blade farther to the right from the CM, thereby increasing the body's inward tilt. These findings suggest that skaters need to greatly tilt the body inward to exert centripetal acceleration in the left stroke and that more than one method can be used to increase the inward tilt of the body.

### 4.3 Practical applications

Our results suggested an overall trend that effective push-off became harder as the knee-joint angle increased and the race progressed, leading to a decrease in the centripetal acceleration during curve skating. However, more skilled skaters were able to skate the curved section in a shorter time by tilting their bodies further inward, thereby increasing the centripetal acceleration in the final stage of the race. Tilting the left shank inward is not the only method for leaning the body further inward during the left stroke.

This study revealed that to shorten the skate time through curved sections, the body should be tilted more inward, and the centripetal acceleration should be increased. Coaches could provide more effective training sessions by paying attention to whether skaters are tilting their bodies as inward as possible. Additionally, the study finding that multiple methods are possible to enable and promote the inward tilting of the body during the left stroke may be useful for coaching skaters who exhibit difficulty in tilting their left shank inward due to skeletal influences.

### 4.4 Limitations

This study has some limitations. First, we analyzed ladies' world-class long-distance speed skaters. Therefore, the results obtained may not be applicable to skaters at lower competitive levels, junior skaters, male skaters due to different body size, or skaters in short-distance events. However, the finding that the body should be tilted more inward and that the centripetal acceleration should be increased to skate through curved sections within a shorter time is one that could be applicable to most speed skaters. Second, we analyzed one stroke each on the left and right, made near the center of the curved section. Therefore, the centripetal acceleration and skating motions occurring at the entrance and exit of the curved section were not clarified. Additional analysis is needed to further explore the factors that contribute to a shorter skating time through a curved section. Third, all data in this study were calculated from video images. Therefore, it was not possible to obtain indicators such as the actual force exerted by skaters or their fatigue. To derive more effective suggestions for training, it will be necessary to conduct further experiments using force sensors and physiological indices.

## 5 Conclusion

In this study, we aimed to identify the factors necessary for shortening the skating time through a curved section by analyzing the centripetal acceleration and skating motions during curving in a 3,000 m race for world-class female long-distance speed skating. The study findings are summarized as follows:

1) The centripetal acceleration and directional velocity decreased as the race progressed.
2) Multiple regression analysis revealed that the centripetal acceleration during the left and right strokes had a significant effect on the curved-section time in the same race stage. Additionally, the directional velocity and curvature radius greatly impacted the centripetal acceleration in the same stroke.
3) As the race progressed, the right blade position became more backward relative to the CM at the end of the right stroke. Furthermore, the left thigh and the right and left shanks were raised, resulting in a posture with a high CM.
4) In the right stroke, skaters with a greater centripetal acceleration exhibited a greater inward tilting of the body and the right shank compared to those with a lower centripetal acceleration. In the left stroke, skaters with a greater centripetal acceleration exhibited a greater inward tilting of the body compared to those with a lower centripetal acceleration but did not necessarily exhibit a greater inward tilting of the left shank.

## Data Availability

The raw data supporting the conclusions of this article will be made available by the authors, without undue reservation.
